# SEOM Clinical guidelines for the treatment of metastatic prostate cancer

**DOI:** 10.1007/s12094-014-1225-3

**Published:** 2014-10-16

**Authors:** J. Cassinello, M. A. Climent, A. González del Alba, B. Mellado, J. A. Virizuela

**Affiliations:** 1 Medical Oncology Service, Hospital Universitario de Guadalajara, Guadalajara, Spain; 2 Medical Oncology Service, Fundación Instituto Valenciano de Oncología, Valencia, Spain; 3 Medical Oncology Service, Hospital Universitari Son Espases, Palma de Mallorca, Spain; 4 Medical Oncology Service, Hospital Clinic de Barcelona, Barcelona, Spain; 5 Medical Oncology Service, Hospital Universitario Virgen Macarena, Seville, Spain

**Keywords:** Androgen deprivation treatment, Castration-resistant prostate cancer, Docetaxel, Cabacitaxel, Abiraterone, Enzalutamide, Radium 223

## Abstract

Androgen deprivation treatment is the current standard first-line treatment for metastatic prostate cancer. For several years, docetaxel was the only treatment with a proven survival benefit for castration-resistant prostate cancer (CRPC). Since docetaxel became standard of care for men with symptomatic metastatic castration-resistant prostate cancer (CRPC), three treatment virtual spaces, for treatment and drug development in CPRC, have emerged: pre-docetaxel, docetaxel combinations and post-docetaxel. Sipuleucel-T, cabazitaxel, abiraterone, enzalutamide and radium-223 have been approved in the pre- or post-docetaxel setting in metastatic CRPC during the last few years. Patients are now living longer and experiencing better quality of life. Strategies for patient selection and treatment sequencing are therefore urgently required.

## Introduction

Prostate cancer is the most frequent urogenital malignancy, the most common solid neoplasm and the second most common cause of cancer death in men in Europe. Age-standardised cases of incidence and mortality per 100,000 population per year were 96.0 and 19.3 in Europe, and 96.8 and 15.2 in Spain respectively [1].

## Standard androgen deprivation therapy

After radical local therapy either with radical prostatectomy or radiotherapy, about 30 % of patients develop advanced disease. Androgen-suppressing strategies have become the mainstay of management of advanced prostate cancer [2] and they can be achieved by suppressing the secretion of testicular androgens by surgical or medical castration or inhibiting the action of circulating androgens using anti-androgens that compete for the androgen receptor in tumoral cells. The standard castrate level of testosterone is <50 ng/mL. Bilateral orchiectomy is a simple and complication-free procedure, except for its negative psychological effect on patients. Long acting LHRH agonists are currently the main form of androgen deprivation therapy (ADT). In a meta-analysis they have comparable efficacy to orchiectomy and diethylstilbestrol [3]

Level of evidence: 1a.

Although based on indirect comparisons, all LHRH agonists seem to be equally effective whatever their formulation is (busereline, gosereline, leuproreline and triptoreline). Anti-androgens compete with testosterone and DHT at the receptor level in the prostate cell. They can be classified as steroidal (cyproterone acetate, megestrol acetate) or non-steroidal (flutamide, bicalutamide, nilutamide, enzalutamide). All forms of castration used as monotherapy (e.g. orchiectomy, LHRH and diethylstilboestrol) have equivalent efficacy.

Level of evidence:1b.

Concomitant administration of an anti-androgen during the first weeks of LHRH agonist treatment is recommended to prevent the effects of testosterone flare-up. Complete or maximal androgen blockade (CAB) consists in the addition of an anti-androgen to an LHRH antagonist. Although it has shown very good preliminary results, meta-analyses and systematic reviews have indicated that CAB appears to provide a small five-year survival advantage, of less than 5 % [4, 5].

Level of evidence:1a.

 Recommendation: All forms of castration used as monotherapy have equivalent efficacy. Long acting LHRH agonists are currently the main form of androgen deprivation therapy. CAB appears to provide a small 5-year survival advantage, of less than 5 %, versus monotherapy.

### When should ADT be initiated?

Controversy exists over the most appropriate time to introduce hormone therapy in patients with asymptomatic advanced prostate cancer, but it is accepted that it should be instituted in symptomatic patients. There is a lack of properly conducted, randomised, controlled trials. Based on a systematic review of the literature, the recently published ASCO guidelines concluded that no recommendation can be made on when to start hormone therapy in patients with advanced asymptomatic PC until data become available from studies using modern diagnostic and biochemical tests and standardised follow-up schedules [6]. The Cochrane Library review extracted four good-quality, randomised, controlled studies which were all conducted in the pre-PSA era. According to the analysis, early androgen suppression significantly reduced disease progression and complication rates due to progression itself, but did not improve cancer-specific survival and provided a relatively small benefit in OS [7].

Level of evidence: 1b.

Recommendations: Immediate ADT (given at diagnosis) significantly reduces disease progression, prevents potentially catastrophic complications, and palliates symptoms effectively, compared with deferred ADT (delivered at symptomatic progression). However, the survival benefit is at best marginal and not related to cancer-specific survival. For asymptomatic metastatic patients, an active clinical surveillance protocol may be an acceptable option in clearly informed patients if survival is the main objective.

### Is intermittent ADT (IADT) standard treatment?

The use of IADT is still controversial. Although EAU guidelines state that its use should no longer be regarded as investigational (Level of evidence: 2), IADT can be used in patients relapsing after radiotherapy and with clear response after the induction period [8]. 

Level of evidence: 1b.

In metastatic patients, a trial of IADT showed worse survival results (SWOG trial 9346) so it cannot be recommended as standard treatment in this clinical setting [9].

### Should ADT be instituted in M0 patients with rising PSA levels?

Although patients with postoperative PSA recurrences often undergo ADT before there is any evidence of metastatic disease, the benefit of this approach is uncertain. Some retrospective studies do not show differences in the time to clinical metastases compared to delayed ADT and do not allow any definitive conclusion on the use of early ADT [10]. In any case, patients without evidence of metastatic disease should not be offered any of the new anti-androgen treatments outside a clinical trial.

### Can chemotherapy be administered in hormonosensitive metastatic prostate cancer?

The results of the Charted study (E3805) were presented at the last ASCO meeting in 2014. This study was performed in 790 androgen sensible metastatic prostate cancer patients who were randomised to receive continuous ADT alone or in association with 6 cycles of taxotere chemotherapy at standard dose. Overall survival was the primary endpoint and results showed a median overall survival of 44 months for ADT treated patients compared to 57.6 month for chemotherapy and ADT treated patients [HR = 0.61 (0.47–0.80); *p* = 0.003]. This difference was even higher in the subgroup of patients with high tumoral volume (32.9 vs. 49.2 months; HR 0.6; *p* = 0.006). Authors conclude that six cycles of chemotherapy in addition to ADT represent an appropriate option for men with mPC commencing ADT who are suitable for docetaxel therapy. These results contrast with those of the GETUG15 trial, performed in a similar population, in which no overall survival differences were found.

## Definition of castration-resistant prostate cancer (CPRC)

CRPC is defined as disease progression despite the administration of androgen suppression therapy. CRPC is a heterogeneous disease and includes patients without metastases with rising PSA only, asymptomatic patients with metastases (mCRPC) and patients with metastases and severe cancer symptoms.

### CRPC definition criteria

The Prostate Cancer Clinical Trials Working Group 2 (PCWG2) defines the following criteria [11]: serum castration levels of testosterone (testosterone <50 ng/dL or <1.7 nmol/L), PSA progression and/or clinical progression to castration. Anti-androgen withdrawal for at least 4–6 weeks is recommended before considering the diagnosis of CRPC.

PSA progression is defined as three consecutive rises of PSA, 1 week apart, resulting in two 25 % increases over the nadir value, with a PSA level >2 ng/mL. Clinical progression includes progression of bone lesions (two or more lesions on bone scan) or soft tissue progression using response evaluation criteria in solid tumors (RECIST).

### Is androgen suppression withdrawal an unavoidable therapeutic and diagnostic procedure?

No. The PCWG2 recommends not to delay new treatment after withdrawal in patients who have not responded to previous treatment, or have shown a decline in PSA levels for 3 months or less when anti-androgens have been administered in second or subsequent treatment lines.

## How to manage asymptomatic or minimally symptomatic mCRPC patients

There are three drugs that have shown to improve overall survival in metastatic castration-resistant prostate cancer (mCRPC) asymptomatic or minimally symptomatic patients in three randomised phase III studies.

### How to define asymptomatic or minimally symptomatic patients

Patients with ECOG Performance Status grade 0 or 1, with a low level of pain as measured by the Brief Pain Inventory-Short Form scale (BPI-SF) 0–1 (asymptomatic) or 2–3 (minimally symptomatic), respectively. Metastatic disease had to be documented by bone lesions on bone scan or by measurable soft tissue disease by CT/MRI.

Drugs that have demonstrated impact on survival in this group of patients are described as follows:

### Sipuleucel-t

Sipuleucel-T is an autologous active cellular immunotherapy which prolongs overall survival among asymptomatic men with mCRPC, with a relative reduction of 22 % in the risk of death, as compared to the placebo group, and an improvement of 4.1 in median survival (IMPACT trial) [14].

Recommendation: Sipuleucel-T is a treatment option in asymptomatic patients with mCRPC before chemotherapy with docetaxel if regulatory approval is obtained in Europe.

Level of evidence: I. Strength of recommendation: A.

### Abiraterone

Abiraterone (AA), an inhibitor of androgen synthesis, in combination with prednisone was superior to placebo plus prednisone in asymptomatic or minimally symptomatic patients without visceral metastasis who had not yet received chemotherapy (COU-AA-302 trial). In an ad interim analysis with 55 % of the required events, overall survival (OS), radiographic PFS (rPFS) and secondary endpoints all favoured the AA arm although only PFS achieved the required level of significance. Abiraterone-prednisone also showed superiority over prednisone alone with respect to time to initiation of cytotoxic chemotherapy, opiate use for cancer-related pain, PSA progression and decline in performance status. Abiraterone has an excellent tolerability profile and the most common side effects were mineral-corticoid effects (edema, hypertension and hypokalemia). Abiraterone has a low incidence of grade 3–4 liver toxicity (8 %) but it is important to monitor liver function, especially during the first months of treatment [15].

Recommendation: Abiraterone is a treatment option for asymptomatic or minimally symptomatic patients with mCRPC without visceral metastases previously untreated with chemotherapy.

Level of evidence: I. Strength of recommendation: A.

The role of abiraterone in groups of patients not included in the COU-AA-302 study, especially symptomatic or visceral metastasis patients, is controversial. In these patients, abiraterone might be an appropriate therapeutic option, especially in patients who are not candidates for chemotherapy (only if clearly not even suitable for weekly or bi-weekly taxotere administration), bearing in mind the benefits in pain palliation and survival demonstrated by abiraterone in symptomatic patients and/or visceral metastases previously treated with docetaxel.

### Enzalutamide

Enzalutamide is a new anti-androgen that has shown benefit in overall survival versus placebo in the PREVAIL phase III study in asymptomatic or minimally symptomatic chemo-naive mCPRC patients. In this study only 4 % of patients had associated corticosteroid treatment and approximately 12 % had visceral metastases (lung and/or liver). The study demonstrated a statistically significant benefit in OS and rPFS of patients treated with enzalutamide compared to those receiving placebo, with a mortality risk reduction of 29 % compared to placebo and a reduction of 81 % percent of the risk of radiological progression or death compared to placebo. Enzalutamide also delayed PSA progression, decline in performance status and time until the first skeletal-related event, and delayed median time to chemotherapy by 17 months, with a favourable tolerability profile (fatigue, back pain, constipation, arthralgia, hypertension) [16].

Recommendation: Enzalutamide is approved for the treatment of asymptomatic or minimally symptomatic patients with mCRPC who have not received chemotherapy.

Level of evidence: I. Strength of recommendation: A.

### Criteria for progression in asymptomatic or minimally symptomatic patients

Periodic radiological assessment using CT/RMI and bone scan is mandatory.

- Radiographic progression is established according to Response Evaluation Criteria in Solid Tumors (RECIST 1.1) or to adjusted PCWG2 criteria.
- The clinical criteria for progression are a significant increase in pain levels and increased analgesic requirements, together with a decline in functional status.
- A PSA rise without evidence of confirmed radiographic progression or skeletal-related events is strongly discouraged as a criterion for starting new systemic antineoplastic therapy, especially in the first 3 months of treatment.

Recommendation: Asymptomatic or minimally symptomatic patients with evidence of rapidly progressing disease should discontinue treatment and be offered other therapeutic alternatives without delay.

Level of evidence: I. Strength of recommendation: A.

## Castration resistant and symptomatic patients

### What is the value of chemotherapy in symptomatic M1 CRPC?

In 2004, two phase III studies demonstrated the superiority of docetaxel with prednisone over the mitoxantrone scheme [12]. Of these two studies the most important was the so-called TAX327, which randomised 1,006 patients to receive docetaxel 75 mg/m^2^ every 21 days, weekly docetaxel 30 mg/m^2^ or mitoxantrone 12 mg/m^2^ every 21 days; all patients received oral prednisone 5 mg administered every 12 h. The group of patients who received tri-weekly docetaxel reduced their risk of death by 24 % compared to the mitoxantrone arm, with a median survival of 18.9 months *vs.* 16.5 months, respectively (HR 0.76, *p* = 0.009). In addition, this group achieved significant benefits in pain improvement (35 vs. 22 %, *p* = 0.01) and quality of life (22 vs. 13 %, *p* = 0.009) compared with the group of patients receiving mitoxantrone [13]. Therefore, the scheme of docetaxel 75 mg/m^2^ in combination with prednisone 5 mg every 12 h became the treatment of choice in patients with symptomatic CRPC.

For patients unlikely to tolerate this 3-weekly scheme due to the presence of co-morbidities, 50 mg/m^2^ docetaxel administered every 2 weeks could be a useful option in this context [17].

### When to start with docetaxel-based chemotherapy in patients with CRPC?

Apart from the presence of symptoms, there are no defined predictive factors to establish which of the patients will benefit from docetaxel-based chemotherapy *vs.* hormone maneuvers, including abiraterone or enzalutamide, in asymptomatic or minimally symptomatic patients. There are general factors that predict a rapidly progressive disease and shorter survival and could justify the choice of docetaxel as first line: the presence of anaemia, multiple metastatic sites, elevated LDH and alkaline phosphatase, and a PSA-DT of less than 55. In these patients, docetaxel-based chemotherapy would be the treatment of choice.

Recommendation: Docetaxel chemotherapy is appropriate for symptomatic patients with metastatic castration-resistant disease and good performance status, and should also be discussed with asymptomatic or minimally symptomatic patients.

Level of evidence: I. Strength of recommendation: A.

## Treatment of mCPRC patients after progression to docetaxel

A range of options is now available, including one cytostatic (cabazitaxel) [18], two hormone treatments (abiraterone and enzalutamide) [19, 20], and one isotope (Radium-223) [21].

The Phase III TROPIC trial demonstrated the efficacy of cabazitaxel plus prednisone vs. mitoxantrone and prednisone, in the treatment of mCRPC, showing a 30 % reduction in the risk of mortality and RR (14.4 vs. 4.4 %, *p* = 0.0005) [18]. The COU-A-301 trial compared abiraterone plus prednisone with placebo plus prednisone, indicating prolonged survival among mCRPC patients with progression disease treated with abiraterone after docetaxel-based chemotherapy (14.8 vs. 10.9 m HR 0.65; *p* < 0.0001) [19]. The AFFIRM study compared enzalutamide versus placebo, and showed that enzalutamide prolongs survival in men with mCRPC after chemotherapy (18.4 vs. 13.6 m HR 0.63; *p* < 0.001) [20]. The ALSYMPCA study compared Radium-223 (alpha-particle emission) to placebo and also demonstrated increased survival rates (14.9 vs. 11.3 m. HR 0.70; *p* < 0.001) [21].

There is no drug of choice because none of these treatments have been compared with each other. Predictive markers must be discovered and validated in order to make rational treatment decisions. Accessibility to drugs, patient co-morbidities and patient and physician preferences can help in treatment decisions. Patients with only bone disease and nodes of less than 2 cm can be considered for Radium-223.

Recommendation: There are several options of choice after docetaxel treatment, according to patient characteristics and drug availability. Strategies for patient selection and treatment sequencing are therefore urgently required.

Level of evidence: I. Strength of recommendation: A.

### Is retreatment with docetaxel possible in any patients?

Retreatment with docetaxel is one of the options available for patients who have responded to first-line docetaxel and who have not progressed while on docetaxel and TTP >5 m [22–24].

Level of evidence: III. Strength of recommendation: C.

### Are there any other chemotherapy treatments?

Mitoxantrone can be used for short-term palliation of symptoms [25].

Level of evidence: IV. Strength of recommendation: D.

## Sequential treatment

### Are taxanes active after abiraterone or enzalutamide?

There are limited clinical and preclinical data suggesting the existence of cross-resistance between docetaxel and enzalutamide or abiraterone. In vitro studies indicate that taxanes may act by disrupting AR signalling. This may represent a potential mechanism for cross-resistance in mCRPC among taxanes and new hormonal agents [26]. However, taxanes also induce cell death through AR-independent mechanisms that may overcome prior hormone-therapy resistance and act against AR-negative cells [27, 28].

### Docetaxel after abiraterone

In a retrospective review on the efficacy of docetaxel in 35 patients previously treated with abiraterone [29], docetaxel showed a 26 % PSA response rate, a median time to PSA progression of 4.6 months and median OS of 12.5 months. In another study, 23 patients treated with docetaxel after abiraterone showed a 48 % PSA response, with a median OS from the date of the first docetaxel dose of 12.4 months [30].

### Cabazitaxel after abiraterone and enzalutamide

A retrospective analysis of 59 men (37 with prior abiraterone and 9 with prior enzalutamide) has been reported. PSA and soft tissue response was observed in 39 and 14 % of patients, respectively, and symptomatic benefit was achieved in 24 % of them. Median OS and PFS were 15.8 and 4.6 months, respectively [31]. Similarly, in a recent published paper, 79 patients treated with third-line cabazitaxel after docetaxel, followed by abiraterone or enzalutamide, showed a PSA decline of 30 %, with a median PFS and OS of 4.4 and 10.9 months, respectively 27. Overall, cabazitaxel seems to remain clinically active despite prior hormone manipulations.

### What is the activity of enzalutamide after abiraterone and docetaxel?

A retrospective analysis of small series of patients reported a response rate that is between 13 and 46 % in patients treated with enzalutamide after docetaxel, with lower treatment duration, PFS and OS that in reported second line randomised trials [31–33]. In one study, the authors reported that prior response to docetaxel and abiraterone did not predict response to enzalutamide [13]. However, another group reported that higher PSA-responses were observed among abiraterone-sensitive patients (44 %) than in abiraterone-insensitive patients (16 %) [33]. Overall, enzalutamide seemed to retain its clinical activity in patients who had progressed to prior abiraterone and docetaxel, and it is not clear whether abiraterone resistance predicts enzalutamide benefits.

### What is the activity of abiraterone after enzalutamide and docetaxel?

A retrospective analysis of series of patients who had progressed following treatment with docetaxel and enzalutamide showed modest activity of abiraterone. One series reported a PSA response of 8 % and a median PFS of 2.7 months [34]. Similarly, other series showed that median abiraterone acetate treatment duration was 13 weeks, median abiraterone time to progression was 15.4 weeks, median OS 50.1 weeks (95 % CI 28.3–72.0) and only three patients had ≥30 % in PSA decline (two of them with prior progression to enzalutamide) [35].

 Recommendations: The true impact of sequential therapy is not yet established. However, based on limited clinical data, sequential treatment has shown to exhibit clinical activity and may be considered in patients with mCRPC (Grade of recommendation C).
